# Tissue adhesives for meniscus tear repair: an overview of current advances and prospects for future clinical solutions

**DOI:** 10.1007/s10856-016-5694-5

**Published:** 2016-03-12

**Authors:** A. I. Bochyńska, G. Hannink, D. W. Grijpma, P. Buma

**Affiliations:** Orthopaedic Research Laboratory, Department of Orthopaedics, Nijmegen Centre for Molecular Life Sciences, Radboud University Nijmegen Medical Centre, Nijmegen, The Netherlands; Department of Biomaterials Science and Technology, MIRA Institute, University of Twente, Enschede, The Netherlands; Department of Biomedical Engineering, W.J. Kolff Institute, University Medical Centre Groningen, University of Groningen, Groningen, The Netherlands

## Abstract

Menisci are crucial structures in the knee joint as they play important functions in load transfer, maintaining joint stability and in homeostasis of articular cartilage. Unfortunately, ones of the most frequently occurring knee injuries are meniscal tears. Particularly tears in the avascular zone of the meniscus usually do not heal spontaneously and lead to pain, swelling and locking of the knee joint. Eventually, after a (partial) meniscectomy, they will lead to osteoarthritis. Current treatment modalities to repair tears and by that restore the integrity of the native meniscus still carry their drawbacks and a new robust solution is desired. A strong tissue adhesive could provide such a solution and could potentially improve on sutures, which are the current gold standard. Moreover, a glue could serve as a carrier for biological compounds known to enhance tissue healing. Only few tissue adhesives, e.g., Dermabond^®^ and fibrin glue, are already successfully used in clinical practice for other applications, but are not considered suitable for gluing meniscus tissue due to their sub-optimal mechanical properties or toxicity. There is a growing interest and research field focusing on the development of novel polymer-based tissue adhesives, but up to now, there is no material specially designed for the repair of meniscal tears. In this review, we discuss the current clinical gold standard treatment of meniscal tears and present an overview of new developments in this field. Moreover, we discuss the properties of different tissue adhesives for their potential use in meniscal tear repair. Finally, we formulate recommendations regarding the design criteria of material properties and adhesive strength for clinically applicable glues for meniscal tears.

## Introduction

Menisci are crescent shaped discs of fibrocartilage with a triangular cross-section, present in duplicate in each knee joint. The menisci have been recognized as crucial structures in maintaining knee joint stability and articular cartilage homeostasis [1–3]. During normal functioning, they are exposed to shear, tension and compression forces and serve a variety of (bio)mechanical functions, such as load bearing, constituting contact area, guiding rotation and stabilizing translation [4–7]. They also play a role in lubrication and nutrition of the underlying articular cartilage surfaces and by that might help to prevent the development of osteoarthritis [1].

The most commonly occurring injuries of the knee joint are meniscus tears. They originate either from acute injuries of the knee joint (e.g. sport, trauma) or are caused by degenerative changes (mostly in case of elderly patients) associated with early osteoarthritis [8]. Irrespective to their origin, they might cause pain, swelling and locking of the joint, and they may ultimately lead to osteoarthritis [9]. Therefore, it is of utmost importance to provide an effective treatment modality and to prevent this negative scenario.

In the past, it was believed that menisci could be removed without any immediate or long-term consequences for the function of the knee joint and consequently a (partial) meniscectomy was performed as a gold standard treatment. In fact, short-term results were very satisfactory. It resulted in instant pain relief and restoration of knee function, but as osteoarthritis develops very slowly, it took decades to find out that even a partial meniscectomy inevitably leads to joint degeneration [10, 11]. In 1975 Krause et al. showed that menisci have a function in load-transmission and stress absorption in the knee and that peak stresses acting on articular cartilage increase after meniscectomy [6]. Removal of only 15–34 % of the meniscus, a partial meniscectomy, will produce a 350 % increase in contact stress and lead to development of degenerative changes and eventually osteoarthritis in a majority of the patients [12]. Ever since, the preferred strategies aim at restoration of the integrity of the meniscus rather than at its removal by (partial) meniscectomy [13]. These treatment methods can only be effective if the torn parts of the tissue are kept in close proximity to each other enabling a healing process to take place. Many devices have been developed and tested in clinical practice for this purpose, such as sutures, stingers, staples, arrows and darts [14, 15]. However, the success rate of these devices highly depend on the location of the meniscal lesion [16]. They are mainly effective in treating lesions located in the peripheral vascularized region of the meniscus [17, 18]. Moreover, the techniques are challenging, surgical procedure is time-consuming and devices are expensive. Therefore, it would be ideal to find an alternative treatment method, which should be easier and faster to perform, and would allow a more universal successful treatment of tears, also those located in the avascular region of the meniscus.

Recently, much interest has been paid to tissue adhesives as an alternative to sutures. Tissue adhesives are already used in clinical practice for other purposes, such as Dermabond^®^ (2-octyl cyanoacrylate) as topical skin adhesive, fibrin glue for pulmonary leaks, and recently TissuGlu^®^ (an FDA approved urethane-based adhesive) for abdominoplasty surgery [19, 20]. However, up to now, there is no clinically available glue suitable for the treatment of meniscal tears.

The purpose of this paper was to summarize current advances in the development of tissue adhesives, compare them with the current clinical gold standard treatments and critically evaluate their potential application in the treatment of meniscal tears. Mechanical and biological properties of the meniscus are discussed and requirements for a suitable material are formulated. Subsequently, characteristics of the main groups of tissue adhesives found in literature are discussed and conclusions and recommendations for future clinical directions are made.

## Biology of the meniscus

**Fig. 1:**
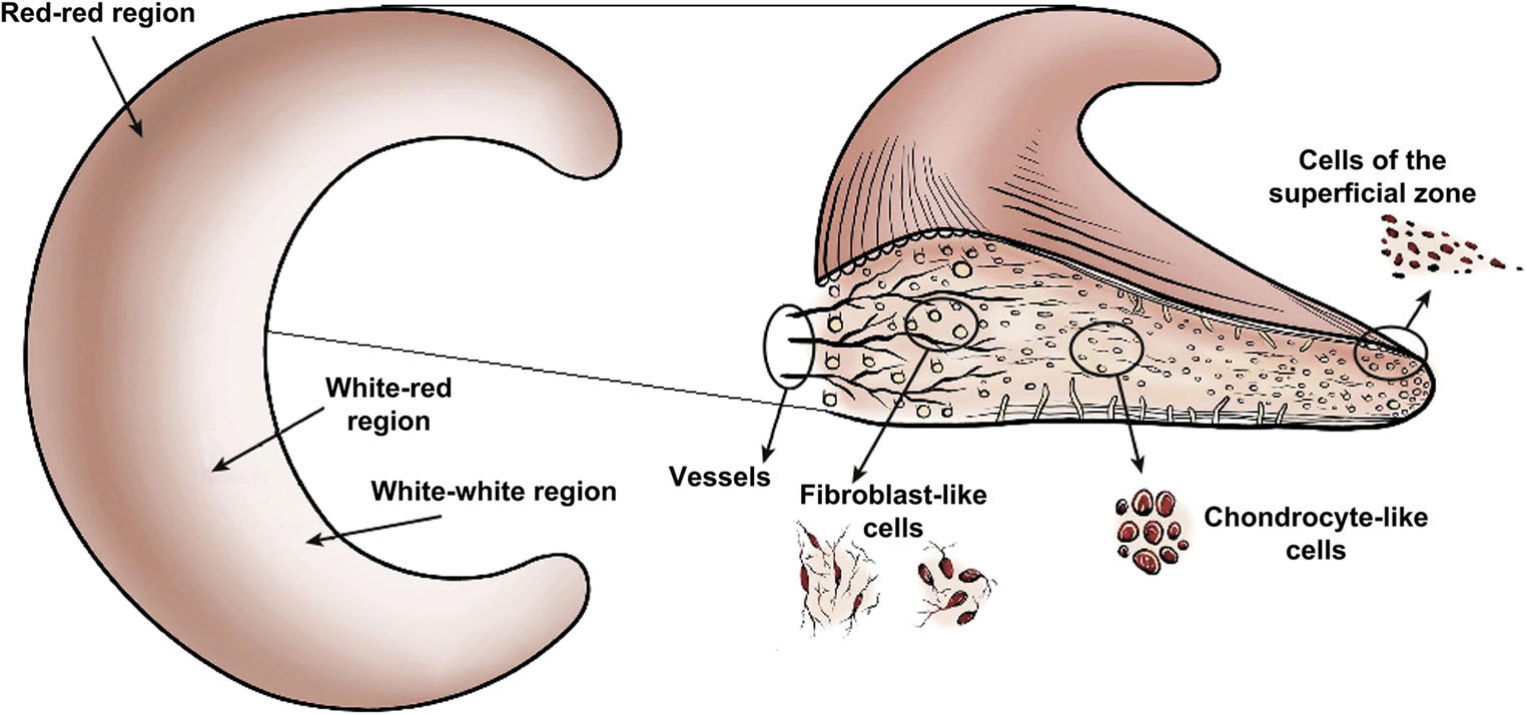
Regional variations in vascularization and cell populations of the meniscus, showing on the *left* the *red–red*, *white–red* and *white–white* regions, and on the *right* the differences in cell populations and the location of the blood vessels. Reprinted from [23] (Color figure online)

The healthy meniscus contains 72 % water, the remaining 28 % are organic compounds: 22 % collagen, 0.8 % glycosaminoglycans (GAGs, of which the major part (40 %) is chondroitin 6-sulphate), DNA (2 %), adhesion glycoproteins (<1 %) and elastin (<1 %) [21]. These proportions may vary depending on age or degenerative status. For instance, with increasing degeneration the water content can increase up to 85 % [21] and the cellularity of the meniscus decreases [22]. The blood supply of the meniscus is also age dependent: the meniscus of an infant is fully vascularized, while an adult meniscus is only partly vascularized, i.e. the outer part. An adult meniscus can be divided into three zones: the outer (red–red), middle (red–white) and inner (white–white) zone, see Fig. 1, [23–25]. The outer two-thirds of the meniscus (red–red and the red–white zone) are vascularized, the cells are fibroblast-like and the collagen fibers present there are mainly type I. The inner one-third of the meniscus (white–white zone) is avascular, contains both collagen type I and II and the cells in this region are more (fibro)chondrocyte-like [26, 27]. Moreover, the inner two thirds of the meniscus contain more GAGs, whose main function is to enable the meniscus to take up water in order to improve their visco-elastic behaviour and counter compressive loads on the tissue [23]. Due to differences in the structure of the meniscus, particularly related to the vascularization and differences in cell phenotype, tears that occur in the outer red–red part of the meniscus have the ability for self-repair. Tears in the middle part are less likely to heal spontaneously, while tears in the inner white–white part are unable to repair themselves [28, 29].

## Biomechanics of the meniscus

**Fig. 2:**
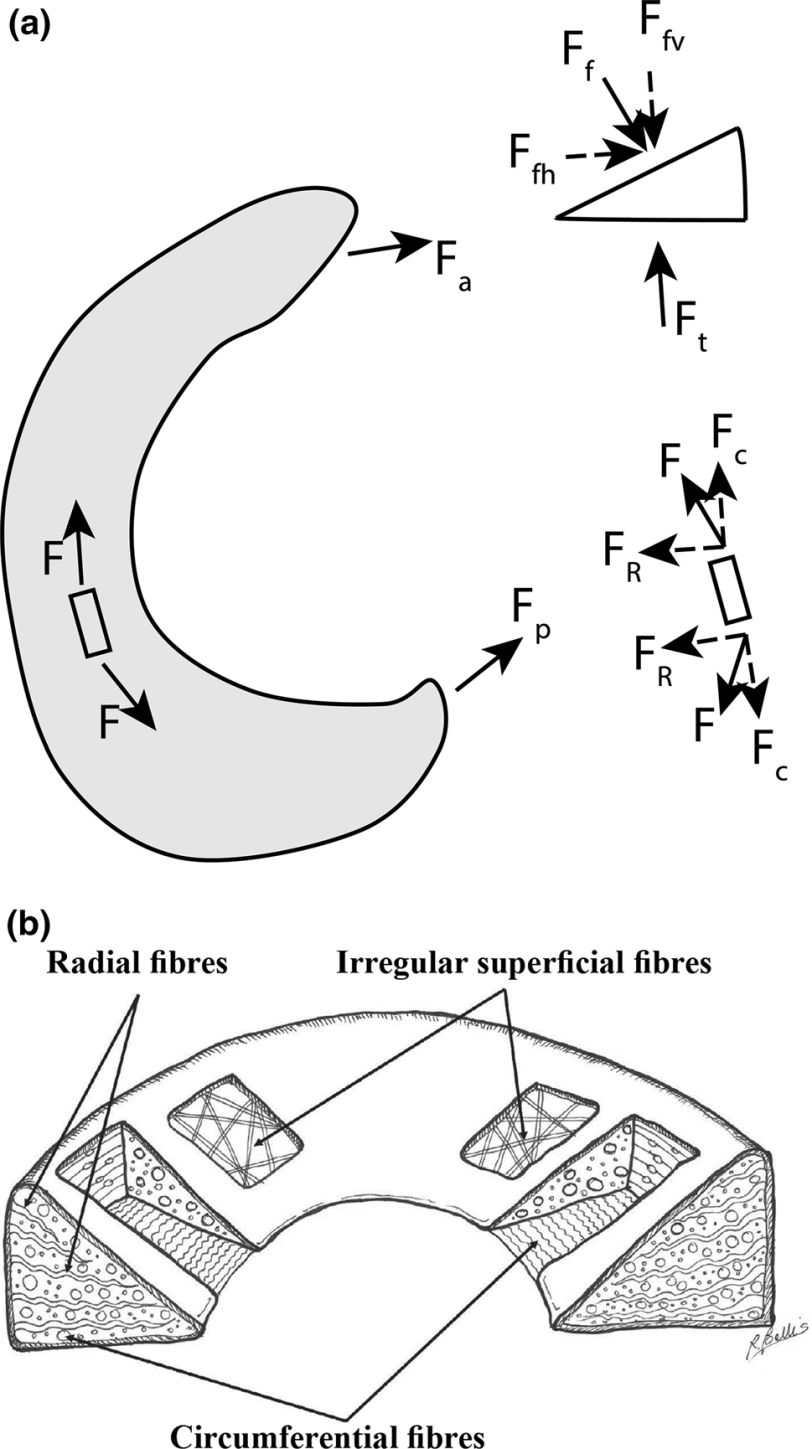
**a** A schematic representation of the forces acting on the meniscus. F_f_ is a joint load on top of the meniscus, F_t_ is the reaction force at tibial plateau, radially oriented force F_fh_ and vertically oriented F_fv_ are balanced by forces generated by anterior and posterior horns F_a_ and F_p_. A combination of these forces results in tensile hoop stress F and axial and radial components of stress in the meniscus (F_r_ and F_c_) while loading [34]. **b** Schematic representation of collagen fibers alignment in meniscal tissue, reprinted from [145]

The two menisci cover an area of approximately 2/3 of the tibial plateau [30]. They stabilize the knee, provide congruity and lubrication of the articular cartilage, and constitute a large contact area between tibial plateau and femoral condyle. The main attachment sites of the medial and lateral menisci in the joint are the menisco-tibial ligamental attachments [31]. Additional ligaments are the transverse ligament and the posterior and anterior meniscus-femoral ligaments (Wrisberg and Humphry) [31]. The medial meniscus is more firmly attached to the joint capsule than the lateral. The lateral meniscus is also more flexible. Throughout the whole range of flexion and extension of the knee joint menisci play a crucial role in transferring loads [32]. They are subjected to tensile, compressive and shear forces and they are displaced radially from the center of femoral condyles due to compressive forces in combination with different flexion angles of the knee [33, 34]. As a result of the compressive loading on the meniscus, the stress is transferred to a vertical force, whose radial component causes a radial displacement of the meniscus. As the meniscus is firmly connected with the tibial plateau through its anterior and posterior attachments, its displacement is constrained by the circumpherentially orientated collagen bundles resulting in a circumpherentially directed component of force and tensile stress (see Fig. 2a), [34]. The collagen fibers in the meniscus structure are organized in a manner that optimally resists these tensile stresses [35]. The surface layer of the meniscus contains sheets of radially oriented collagen fibres, to cope with shear forces, while the deep zone has circumpherentially organized collagen fibres, to withstand the circumpherential forces (see Fig. 2b).

**Fig. 3:**
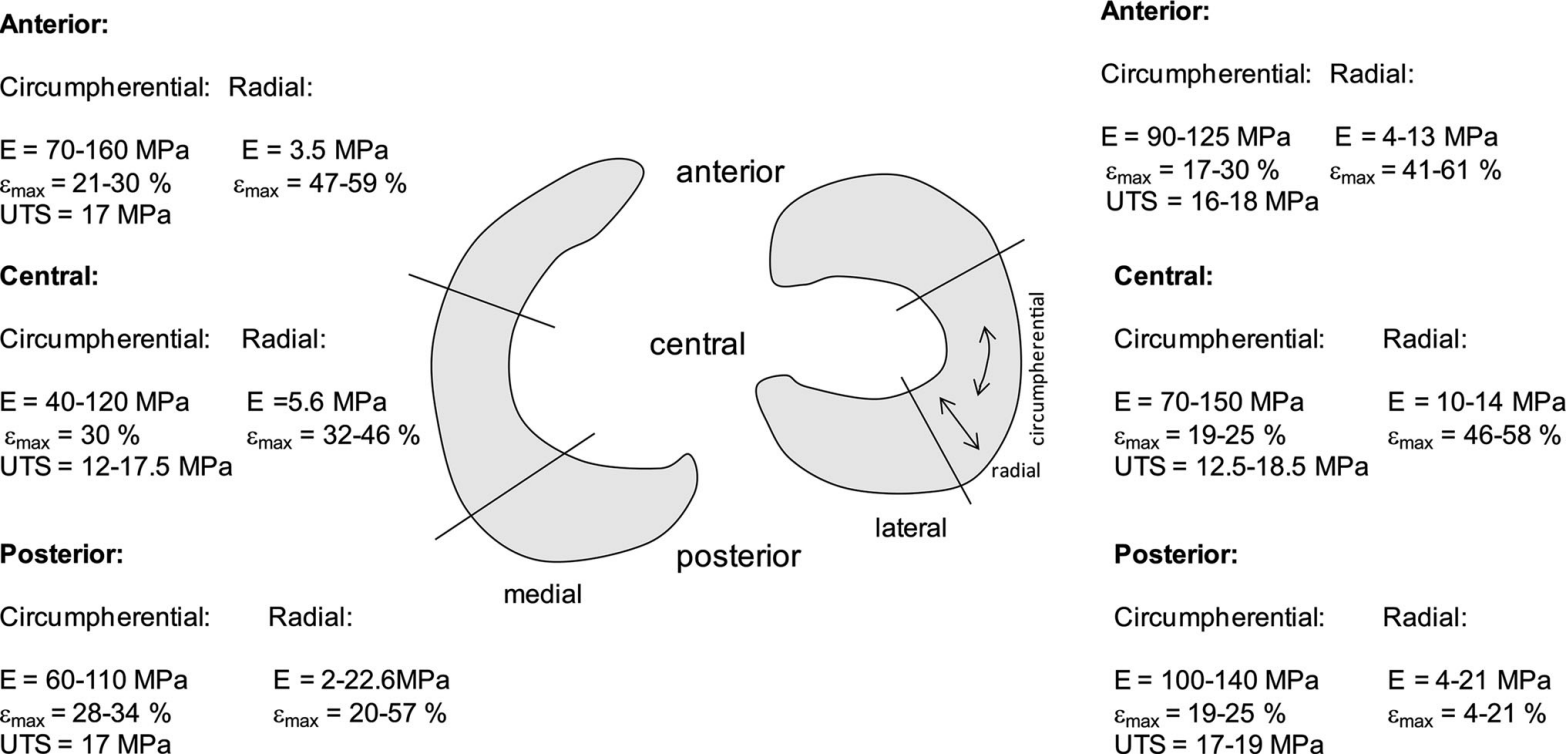
Overview of the tensile properties of anterior, central and posterior part of the human medial and lateral meniscus, based on [35–37]. View on the right knee. *E* elastic modulus, *ε*_*max*_ elongation at maximum applied force, *UTS* ultimate tensile strength (only in circumferential direction)

Biomechanical properties of meniscal tissue (see Fig. 3) have been extensively described in literature. Fithian et al. reported that the tensile modulus measured in circumferential direction was in the range 100–300 MPa and in radial direction between 10 and 30 MPa. The shear modulus of the meniscus was measured to be approximately 120 kPa [35]. Lechner et al. and Tissakht et al. reported a tensile modulus in circumferential direction ranging from 40 up to 140 MPa [36, 37].

## Meniscal tears and their healing potential

**Fig. 4:**
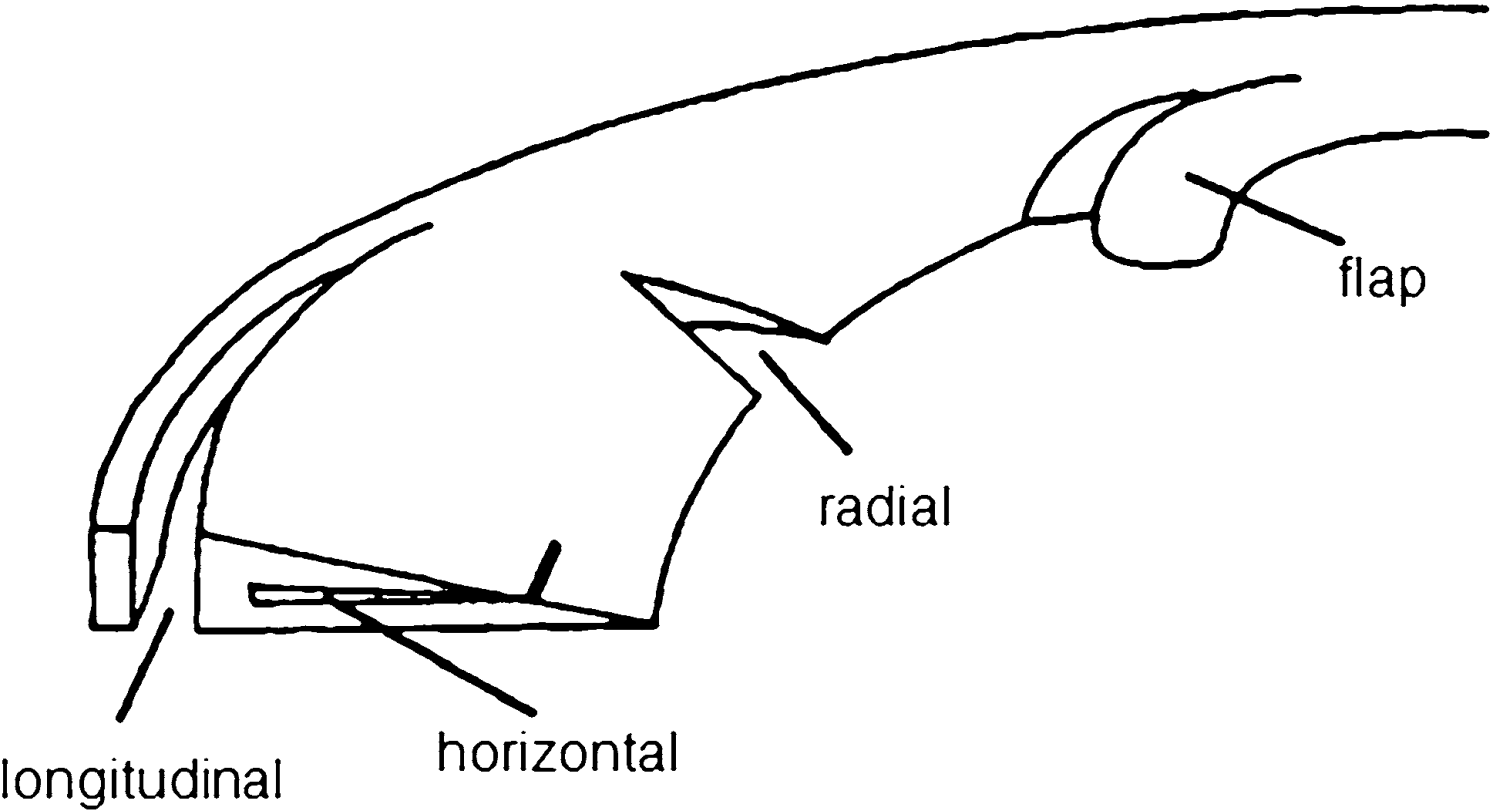
Classification of meniscal tears. Reprinted from [38]

There are two main causes of meniscus tears: trauma (e.g. sports injuries) mostly occurring in young and active patients, and degeneration, which are mainly associated with early stages of osteoarthritis and are more frequently observed in elderly patients [38]. An overview of the classification of meniscal tears is shown in Fig. 4. Longitudinal tears, such as commonly occurring bucket-handle lesions, are classified as traumatic, whereas flap, horizontal and tears in menisci with deteriorating changes are classified as degenerative. Radial tears may be classified both as traumatic and degenerative [38]. Unfortunately, the tears are usually located in the highly loaded, avascular, inner region of the meniscus and do not heal spontaneously. Moreover, it has been reported that there are significantly better results after treating traumatic than degenerative lesions [10]. This is mainly due to the fact that degenerative tears are often associated with early stages of osteoarthritis and disturbed homeostasis of the knee joint, thus in this condition it is more difficult to successfully repair them [39]. The orientation of the tear also influences its ability to heal. Tears that disrupt the circumpherentially oriented collagen fibers may be more difficult to heal or do not heal at all since they interfere with the circumpherential mechanical properties of the meniscus tissue [40–42]. Thus, longitudinal tears are relatively the easiest to heal and the native mechanical function will be restored after healing [29, 43–45]. Therefore, as traumatic lesions are usually oriented in the circumpherential direction, only in these cases in young patients, restoration of the native tissue is the preferred treatment. Elderly patients with degenerative changes are offered alternative treatment methods, such as partial meniscectomy [29], allograft transplantation or implantation of a permanent implant, however these methods are not covered by this review, and are widely described elsewhere [46].

## Current clinical treatment methods of meniscal tears

There are numerous techniques used in clinical practice to restore the integrity and function of torn menisci, most used are sutures, staples, stingers and screws [47]. These devices are employed to keep the torn meniscus together enabling tissue healing. Among these techniques, suturing is superior, since it provides optimal mechanical stability and has the highest strength at failure [8, 11, 47, 48]. Different suturing techniques are used: all-inside, outside-in, inside-out, in order to provide the most suitable treatment for particular tears [49]. The use of vertical sutures provides the best results and is therefore the current gold standard [50]. An alternative to sutures, which are usually difficult to apply during minimally invasive surgery as the procedure is time-consuming and may induce scarring and cause problems with healing, might be using Meniscus Arrows^®^ or Smart Nails^®^, which are easier to apply [51]. However, they carry the risk of chondral abrasion, due to the fact that a part of these devices remain exposed at the surface of the meniscus and harm articular cartilage [52]. In addition, these implants might fail, and as they are usually prepared from non-degradable materials, articular cartilage might be damaged due to the long-term presence of material debris in the knee joint.

In clinical practice, procedures aimed at increasing the rate of healing in the avascular zone are also proposed. Rasping the tissue to expose collagen fibers and induce vascularization, trephination and creation of access channels to facilitate new tissue ingrowth have been employed [53–57]. However, the success rate is found to mainly depend on the type and location of the tear.

## Advances in research of meniscal tears repair

Recent advances in meniscus research suggest that the low cellularity, dense ECM and poor vascularity together with the inflammatory condition of the knee joint in general might also be responsible for the lack of healing potential. Reprogramming of the wound environment is one of the potential targets to increase the healing potential of the meniscus, but this requires extra steps in treating the tissue adjacent the tear [58]. Amongst the procedures which were proposed are the delivery of therapeutics by biomaterial-based technologies and the partial digestion of the matrix in the torn region by collagenase which might enable the migration of cells to the wound margin and stimulate healing [59, 60]. On the other hand an approach in which the level of proteolytic enzymes is controlled may also improve meniscus capacity to repair, as the present inflammation is associated with increased synovial inflammation and increased level of the proteolytic enzymes, especially metalloproteinases (MMP) [61, 62]. In an in vitro repair and animal models it has been shown that by introducing inflammatory cytokines, such as interleukin-1, the integration of the meniscus can be reduced, while inhibiting MMPs and catabolic cytokines can restore its integration capability [63–65]. Another approach which was already proven to have a beneficial influence on meniscal repair is the injection of progenitor cells into the joint, a procedure which enhances tissue regeneration. Also growth factors, especially TGF-β, have been shown to increase proteoglycans synthesis by fibrochondrocytes from different zones of the meniscus in an in vitro cultures [66–68]. Further development of these techniques to clinical use could improve the healing capacity of the meniscus, potentially making the whole healing process shorter and more efficient.

In the light of the complexity of the biological modifications developed to improve the meniscus repair capacity, there might also be a great potential in the use of tissue adhesives. They could serve as an alternative or support for sutures, and would keep the wound edges of the tear close to each other. The use of slowly resorbable tissue adhesives would have numerous advantages, such as easy and fast application, no need of removal after the tissue is healed, and at the same time they could act as a carrier to deliver cells, growth factors, enzymes and other biological factors to the meniscus. Thus, they could be used in combination with the treatment modalities described in the previous section. There already have been attempts in the clinic to use fibrin gels in meniscus repair treatments, either as a glue or as a support to suturing [49, 50, 69–72]. More details of these procedures are given later. However, until now they have not been adopted in clinical practice. It remains a challenge to develop a tissue adhesive which has all the required properties for adequate meniscal tear repair. In this review we focus on a discussion on the desired properties of glues for meniscal tear repair and how current developments in the field comply with them.

## Requirements of tissue adhesives for use in meniscal repair

Tissue adhesives are defined as materials capable to attach and remain on surfaces of biological substrates with ability to interact with biological factors [73]. Any tissue adhesive, regardless its intended application, should fulfil a large number of requirements to be suitable for biomedical use [74]. Most importantly, good attachment to (wet) tissue surfaces is a prerequisite. The material must be non-cytotoxic and not induce any adverse tissue reaction. An adhesive should initially be in a liquid state with an adequate viscosity to be able to apply it only locally on the tissue in adequate amounts. There should be no need for elaborate application techniques or devices, and the adhesive should preferably be injectable. It should wet and spread over the entire surface of the tissue to be glued, resulting in effective and close contact between adhesive molecules and the surface of the tissue. Thereafter, it should solidify shortly after its application [75]. The adhesive should achieve a stable union between the tissue surfaces until proper healing has occurred. To allow this, it must withstand forces present on the site of application. Finally, it should degrade and potentially be resorbed by the body so that the two edges of the wound can reunite to accomplish complete wound healing. Biodegradation products of the cured tissue adhesives must be not cytotoxic and must have sizes allowing their clearance by the kidney (maximally 50 kDa [76]). Moreover, the tissue adhesive should be shelf-stable and preferably coloured for easy visualization [74]. Abovementioned requirements are critical for every tissue adhesive material, regardless of the site of application. For materials intended for specific purposes, additional criteria should be formulated [77].

To formulate specific design criteria for tissue adhesives for meniscus tear repair, the particularity of the tissue in terms of its structure, vascularisation, healing potential and its performance in loaded conditions must be considered. The adhesive has to spread well on the wet surfaces of the meniscus in a synovial environment. Subsequently, it should cure forming a solid network strong enough to withstand the forces present in the knee joint. Therefore, it should be sufficiently hydrophilic to facilitate spreading on the tissue. Secondly, in order to obtain a strong network after curing, the adhesive should preferably contain hydrophobic components as well. These components will contribute to good mechanical properties of the network after curing. The tensile modulus of the cured network should be in the same range as that of meniscus tissue in the circumferential direction, as the majority of injuries fitting criteria for repair occur in the circumferential direction [78]. This means that it should be in the range of values between 40 and 150 MPa (see Fig. 3). The ultimate tensile strength (UTS) of the adhesive should be either higher or comparable to that of meniscus tissue, which is approximately between 12 and 19 MPa in circumferential direction (see Fig. 3).

When designing the degradation profile of the adhesive, the healing rate of the meniscus must be taken into account. In general, patients are advised to use crutches for 8–10 weeks post-surgery after repair of a meniscal tear using sutures to avoid overloading the meniscus tissue during its healing process [29]. At the same time it has been reported that visual evidence of healing of a meniscus requires a 4 month time interval [79]. Therefore, it would be beneficial if the tissue adhesive remains functional at the site of application for at least 4 months before it starts to degrade without rapid loss of its mechanical properties. Finally, the adhesive strength of the adhesive material should be sufficient to keep the torn meniscus together during this period of healing. However, it is hard to determine what exactly should be the adhesive strength due to variability of lesions, intended technique to be used and specificity of each patient’s case. If referred to the mechanical strength of the currently used devices for meniscus repair (sutures, stingers, screws), those depend not only on the device itself but also on the fixation method.

Rimmer et al. compared the failure strengths of 3 arthroscopic meniscal suturing methods [80]. Depending on the technique used (a single horizontal loop, a double vertical loop, and a single vertical loop), the failure strength was between 29.3 and 67.3 N. Barber et al. compared the performance of sutures with other surgical devices [15]. The load to failure was measured of e.g. double vertical stitch (113 N), single vertical stitch (80 N), BioStinger (57 N), a horizontal mattress stitch (56 N), a meniscus arrow (33 N) and the Biomet staple (27 N). It is evident that the variation in mechanical failure strength is broad, and the use of a particular method strongly depends on the individual case, type of lesion and the number of sutures used. Moreover, it is impossible to directly relate tensile strength of sutures with the required adhesive strength of the tissue glue. In studies analysing forces on sutured menisci (both lateral and medial) in human cadaver knee models, it was reported that the distraction forces did not exceed 5 N on average when the applied load was 300 N [81, 82]. In another study, where the distraction forces were assessed indirectly on bucket-handle lesion, the occurring forces were determined to be less than 10 N [83]. These forces would correlate to a stress of 50–100 kPa for a tear measuring 1 cm^2^, and in the non-weight bearing recovery period these forces would probably be even lower. Therefore, tissue adhesives should be designed to resist physiological stresses and by that stabilize the meniscus and prevent gap formation [84]. An adhesive material able to hold a torn meniscus together under a stress of 50–100 kPa should therefore be already sufficient for this application.

Another important aspect that should be taken into consideration in the design of a tissue adhesive for meniscus repair is the incorporation of cells or growth factors. These could enhance healing of the meniscus, especially in the inner a-vascular and a-cellular zone. The tissue adhesive can serve as a carrier for the molecules, which can be released on the site of application e.g. via diffusion process or, if the factors are bound to the glue, they could be released during degradation of the adhesive. The beneficial influence of incorporating both cells [85] and other biological compounds, such as bone marrow or platelet rich plasma (PRP) [84, 86], on meniscus healing have been already reported in literature.

A summary of the most important requirements of tissue adhesives to be used in the repair of meniscus tears is depicted in Table 1.

**Table 1:** Summary of the key requirements of tissue adhesives for meniscus repair

Tissue adhesive		Cured adhesive network		
Application	- Application via syringe/needle (adequate viscosity) - Can be applied via minimally invasive arthroscopical procedures - Easy spreading on the tissue surface (sufficiently hydrophilic), but does not leak to surrounding tissues	Mechanical properties	Elastic modulus	40-150 MPa
			UTS	12-19 MPa
		Biodegradation	-Starts to gradually degrade after 4 months of application -Degrades to non-toxic, low molecular weight products (max 30-50 kDa), which can be renally excreted	
Curing rate	-Cures within few minutes after application allowing surgeon to precisely place it in the tear			
		Biocompatibility	-Does not induce chronic inflammation -Non-toxic degradation products	
Adhesive shear strength	At least 50 kPa			
Biocompatibility	-Not cytotoxic, does not induce acute inflammatory response			

## Current developments in the field of tissue adhesives

Recently, there has been much interest in the research field of tissue adhesives. Besides a few products which are already approved for clinical use (e.g. Dermabond^®^, Fibrin glue, TissuGlu^®^), most research is directed towards the development of new adhesive biomaterials and improvement of the properties of existing ones.

In general, the biomaterials used for the synthesis of glues might be composed of natural, synthetic or combination of both types of polymeric materials [87]. Synthetic biomaterials are usually cheap and their ground substances are widely available, the mechanical and physico-chemical properties might be easy to control, but they can be toxic and may have a sub- optimal biocompatibility. Natural materials, on the other hand, offer better biological properties such as biocompatibility and good cell adhesion, but the production is more difficult to standardize due to variability of the ground substances, they are expensive and carry a risk of disease transmission [88]. In terms of mechanisms of curing and adhesion to the tissue, the materials can be divided into several subcategories. Since numerous reviews were published recently on the chemistry and mechanisms of tissue adhesion [89–91], here only a brief description of the basic mechanisms and examples from literature are outlined.

### Natural protein-based tissue adhesives

#### Fibrin adhesives

**Fig. 5:**
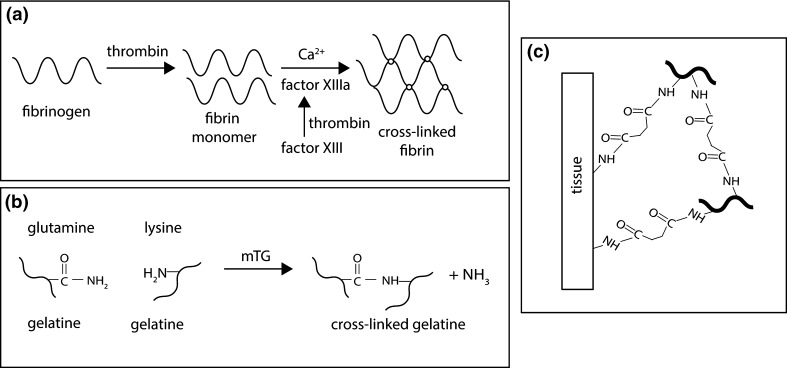
**a** Schematic for fibrin glue cross-linking mechanism [94], **b** cross-linking mechanism of gelatine with calcium-independent microbial transglutaminase (mTG) [94], **c** adhesion mechanism of transglutaminase-cross-linked gelatine to the tissue [146]

The most widely used natural tissue adhesive is fibrin glue (commercially available Raplixa, Artiss, Evarrest, Tisseel). It is a mixture of fibrinogen and thrombin and its curing mechanism mimics the final stages of blood coagulation [77], (Fig. 5a). Fibrin binds to the tissue by three modes: covalent bonding, hydrogen- and electrostatic bonds and mechanical interlocking [92]. Other similarly working adhesives are FloSeal and Proceed (a combination of bovine thrombin and bovine collagen that form the matrix for the clot) and CoStasis, which is a combination of autologous human plasma obtained from patients’ blood and a mixture of bovine collagen and thrombin. The use of fibrin gels in meniscal tear repair has been already described in literature. Ishimura et al. reported results of a study where meniscal tears of 40 patients using a purified fibrin glue were repaired [69]. Within the follow up period of up to 11.4 years post intervention, the rate of recurrence of tears in the red–red zone or red–white zone was below 10 %, whereas that of tears in the white–white zone was 17 %. In another study, Henning et al. repaired a series of meniscal tears using an exogenous fibrin clot [47]. It was reported that in 64 % of cases meniscus was healed. Van Trommel et al. reported on outside-in repair of a radial tear of the lateral meniscus using an exogenous fibrin clot in 5 patients. All 5 menisci were found to have healed on follow-up arthroscopy [72]. In a more recent study, Ra et al. described the use of a fibrin clot together with 2 sutures in the repair of radial meniscal tears in 12 patients [50]. Follow-up MRI performed after 11 ± 3 months postoperatively, revealed complete healing in 11 patients (and incomplete healing in 1 patient). Also recently, Kamimura et al. reported the use of fibrin glue together with sutures in the treatment of degenerative horizontal tears in 18 patients [93]. The follow-up results of 10 patients showed that complete healing was achieved in 70 % of cases. Nevertheless, despite successful repair results of meniscal tears reported in literature, the use of fibrin has not been widely adopted in clinical practice.

#### Gelatine adhesives

Gel-forming mechanisms similar to blood coagulation have been further investigated. Liu et al. [94] proposed that gelatine (as a structural protein) and calcium independent transglutaminase (mTG) (as a cross-linking enzyme) can be used (Fig. 5b). They reported that gelatine-mTG adhesives gelate in situ within few minutes (<5), adhere to tissue in the presence of blood via covelent bonds (Fig. 5c) and provide sufficient strength to be used as a surgical sealant. Also Chen et al. investigated the use of gelatine-mTG adhesives [95]. The materials did not induce structural damage of the retina after injection into the vitreous cavity in rats and their adhesive strength to bovine retinal tissue in vitro was between 15 and 45 kPa. Moreover, peptide-conjugated polymer hydrogels formed by tissue transglutaminase cross-linking were evaluated as an adhesive on guinea pig skin and collagen membrane [96]. The results show that the adhesive strength to the tissue was comparable to that of fibrin glue.

Other adhesives of this class are gelatine–resorcinol–formaldehyde (GRF) and gelatine–resorcinol–formaldehyde–glutaraldehyde (GRFG) glues. The gelatine is cross-linked by aldehyde via reaction with its amine groups. At the same time, aldehyde groups form covalent bonds with amines from the tissue. These materials have been utilized in aortic dissections, liver surgeries, gastrointestinal and urinary track operations [87]. However, safety issues have arisen regarding their toxic degradation product—formaldehyde.

#### Albumin adhesives

**Fig. 6:**
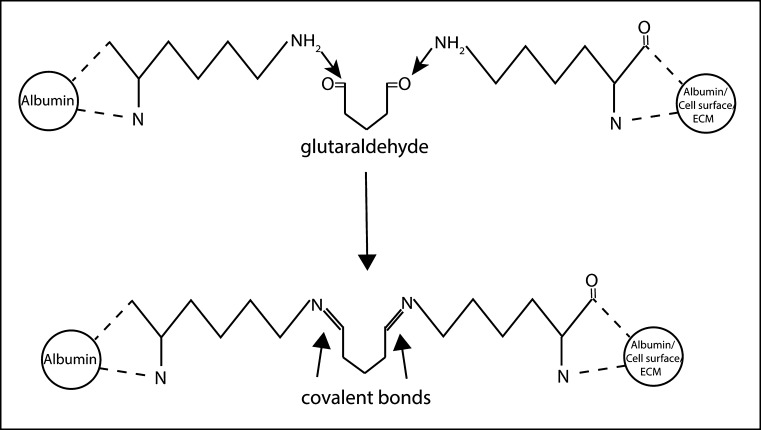
Cross-linking of a commercially available albumin-based adhesive (BioGlue^®^)

In addition, albumin has been investigated as a structural protein for tissue adhesive compositions. Currently BioGlue, one such adhesive that comprises bovine serum albumin (45 %) and glutaraldehyde (10 %) (Fig. 6), is approved by the FDA for the repair of aortic dissections [97]. It closes the cavity of the aorta and provides a stronger arterial wall for its repair, it binds within 30 s and reaches its maximum bonding strength after 3 min [98].

Although natural protein-based cross-linked adhesives have excellent biocompatibility, their bonding strength to the tissue is relatively low, usually in the range of 10–40 kPa. After curing they form networks with the relatively low mechanical properties of soft hydrogels, which limit their application in load bearing sites in the body. Moreover, they are rapidly degraded by enzymes from the body. Normally they degrade within 2 weeks [99], which is too fast for meniscus repair. These glues are however applicable for pulmonary leaks, for haemostasis in vascular surgery and for treatment of cerebrospinal leaks.

### Nature-inspired (biomimetic) tissue adhesives

**Fig. 7:**
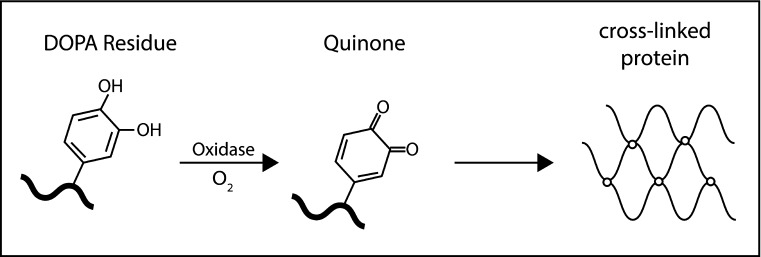
Working principle of a mussel adhesive [147]

Nature-inspired adhesives, for instance based on glues secreted by marine sessile organisms (mussels, barnacles, tube worms), perform very well in hydrated conditions. The working mechanism is depicted in Fig. 7. Mussels enzymatically oxidize the phenolic residues of their adhesive proteins. Then, the oxidized residue undergoes a crosslinking reaction that results in the formation of a 3-dimensional polymeric network. The attachment to the tissue takes place by bonding to amine and thiol groups present on the tissue surface [74]. Due to their high adhesion strength and ability to adhere to wet surfaces, these glues have been proposed for use in biomedical applications. These properties are mainly due to the presence of a redox functional group: 3,4-dihydroxyphenyl-l-alanine (DOPA). The key element of DOPA is ortho-dihydroxylphenyl (catechol), which exhibits strong affinity towards organic surfaces [100]. Therefore, development of biomimetic adhesives have received much attention these last years and artificial materials that mimic natural forms, such as co-polypeptides containing DOPA [101], poly(ethylene glycol) hydrogels and Pluronics functionalized with DOPA, have been synthesized [102, 103].

Another similar mechanism is present in red and brown algae, which also, like mussels, excrete phenolic compounds that can bind to both hydrophobic and hydrophilic surfaces in aqueous conditions. The produced polyphenols are activated by a vanadate-peroxidase type of enzyme that allows the cross-linking of the polyphenol with the extracellular carbohydrate fibers, which will finally lead to adhesion to a surface [104]. Natural algal-borne polyphenol can be successfully replaced by phloroglucinol. Formulations composed of phloroglucinol, alginate and calcium ions are capable of adhering to a variety of surfaces including porcine tissue [105].

One of the strategies to induce a better and stronger interaction with a surface is the increase of the contact area. Geckos have used this in an interesting adhesive strategy that relies on foot pads composed of keratinous foot-hairs that are split into terminal spatula of a size of 200 nm [106]. The adhesion mechanism is based on capillary forces and van der Waals interactions. Inspired by this natural phenomena, Geim et al. developed an adhesive composed of dense arrays of flexible pillars to ensure their collective adhesion. However, problems related to their durability were encountered [107]. To resolve these problems Lee et al. developed an adhesive inspired by mussels and geckos, which combine excellent properties of both mechanisms, and can bind to the surfaces both in dry and wet environment in multiple cycles. The adhesive force in a wet environment was calculated to be equivalent to 90 kPa [108]. Szomor and Murrel tested a naturally-sourced glue secreted by Australian frogs (genus *Notaden*) on sheep meniscus tissue and reported that the adhesive strength of this glue (~90 N) is superior both to fibrin glue (5 times stronger) and gelatine (2.5 times stronger) [109].

Based on recent literature, it can be concluded that biomimetic tissue adhesives have the potential to be used in numerous applications. In general, the adhesive strength of this class of materials is reported to be 20–90 kPa [103, 108, 110]. The cytocompatibility is dependent on the amount of material added to cells, but in vivo tests showed no adverse tissue reactions [103]. However, there is almost no information about degradation profiles of the reported adhesives and of the mechanical properties of the cured networks. Therefore, it cannot be indisputably concluded that these materials could be suitable for treating meniscal tears.

### Synthetic and hybrid tissue adhesives

Another group of adhesives are polymers, natural, synthetic or combination of both, whose end-groups have been modified in a way that allows simultaneous curing and attachment to the tissue. Examples of this class are cyanoacrylates, materials containing *N*-hydroxysuccinimide (NHS)- or isocyanate-terminated polymers, as well as polymers that can be photo cross-linked or those forming networks due to physical interactions e.g. due to thermal gelation.

#### Cyanoacrylate adhesives

**Fig. 8:**
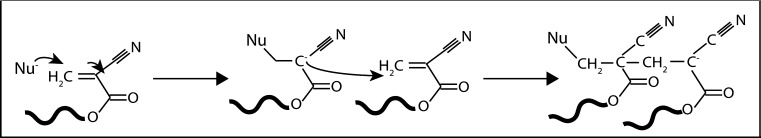
Schematic illustrating the polymerization principle of cyanoacrylates

Cyanoacrylate adhesives are very attractive materials for tissue fixation due to their high bonding strength and ability to bond in a wet environment. The cyanoacrylates monomers polymerize through contact with water (hydroxide ions) or a weak base, such as cell membranes and tissue [111], (see Fig. 8). They are applied to the wound site as cyanoacrylate monomer that undergoes immediate polymerization to form a polymer film, [112]. However, cyanoacrylates still have some serious drawbacks for biomedical application. They degrade reactively in aqueous media with toxic formaldehyde as their degradation product [113, 114]. They are brittle and rigid with the values of elastic modulus in the range of 500–1500 MPa [115]. Additionally, cytotoxic effects may occur due to oxidative conversion of membranous lipids [116]. Nevertheless, clinical use of cyanoacrylate adhesives has been reported as a good replacement for sutures, because of its better cosmetic effect, reduced pain and recovery period [117].

Commercially available Dermabond^®^ and Histoacryl^®^ are widely used as topical skin adhesives, but they are not bio-absorbable and therefore in clinical practice they are used only for skin wounds [97]. Still, the use of Histoacryl^®^ has been compared with sutures in delaying the formation of a 2 mm meniscal gap [118]. The bovine menisci were placed in a tensile loading machine and the force needed for gap formation was measured. The tear was repaired with either vertical sutures, Histoacryl^®^ or both. It was found that Histoacryl^®^ gluing is superior to vertical sutures regarding gap delaying. However, the best results were obtained when glue and sutures were used together. Reckers et al. reported an in vivo study of cyanoacrylate surgical adhesives used for the fixation of transplanted menisci in rabbits [119]. It was concluded that the glue induced necrosis from the cortex to the bone marrow of transplanted bone surface. In order to improve the biocompatibility and mechanical properties of cyanoacrylates, Lim et al. mixed cyanoacrylate with poly-l-DOPA [120] in order to improve the physiochemical properties of cyanoacrylate for use as tissue glue. Lim and Kim also proposed to mix the cyanoacrylate component with poly(l-lactide-*co*-caprolactone) to obtain a biodegradable elastomer [121]. They reported that, although the adhesive strength was decreased compared to a pure cyanoacrylate compound, the biocompatibility and flexibility were improved. This indicates that these materials are more suitable for specific biomedical applications.

In conclusion, of all known adhesives, cyanoacrylates are the class of adhesives that form the strongest bond with tissue. Their adhesive strength values, depending on the tissue type and type of test performed, are reported to be in the range of up to a few MPa (30 kPa adhesive strength to skin up to 2 MPa to bone) [86, 122, 123]. So far, their toxicity limits their application within the body and they have been FDA-approved only for topical skin wound closure. Moreover, their high stiffness (high E modulus), limits their application in soft tissues, such as meniscus.

#### Chemically cross-linkable adhesives

**Fig. 9:**
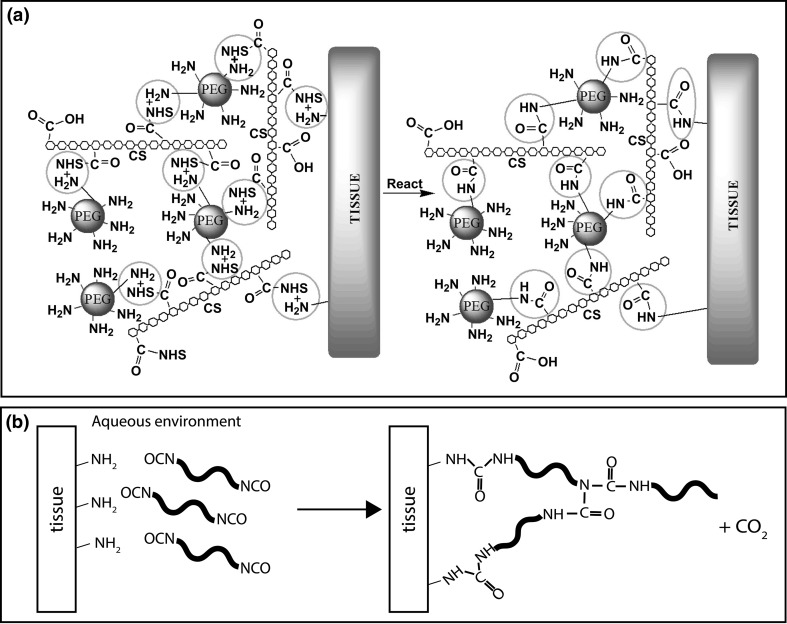
**a** Scheme of the adhesion and network formation mechanism of CS-PEG hydrogels. These hydrogels attach via covalent bonding of the NHS-activated carboxyl groups to the amine groups of the tissue (reprinted from [124]). **b** Scheme of the attachment of isocyanate-terminated polymer molecules to the surface of the tissue by covalent bonding and the formation of a polymer network by cross-linking

In the first class of these materials, the presence of *N*-hydroxysuccinimide activates carboxylic groups and allows them to react with amine groups present on the surface of the tissues to form amide bonds. In contrast, an unactivated carboxylic acid group would just lead to salt formation with an amine. Strehin et al. characterized chondroitin sulphate-PEG adhesive hydrogels (CS-PEG), which covalently adhere to tissue through an amide bond. CS-NHS was mixed with PEG-(NH_2_)_6_ to tune hydrophilicity and the molecules react to form a hydrogel, but at the same time, CS-NHS also reacts with tissue [124], see Fig. 9a. Simson et al. described an adhesive hydrogel composed of a mixture of bone marrow aspirate and chondroitin sulphate end-functionalized with NHS to bind and stabilize the interface of bovine meniscus tissue [84]. The results showed that the adhesive strength to meniscus was in the range of 60–335 kPa, which should be enough to keep a torn meniscus together. Moreover, the viability and proliferation of fibrochondrocytes was positively influenced by the glue. Therefore, it was concluded that this system could be used to mechanically stabilize a tear and stimulate regeneration of the tissue across the injury site.

Taguchi et al. [125] investigated biodegradable adhesives composed of human serum albumin (HSA) and organic acid-based cross-linkers (trisuccinimidyl citrate, disuccinimidyl tartarate (DST) and disuccinimidyl malate) with activated NHS groups. The best results were obtained using DST. The adhesive strength of these biocompatible and bioresorbable materials was better than that of fibrin glue. The active ester groups present in DST react with the amine groups from HAS and collagen molecules from the tissue. The adhesive composed of DST/HSA was evaluated in the gluing of meniscal tears [126]. Glued and sutured porcine menisci were implanted subcutaneously in rabbits, and after 3 months, tensile tests and histology were done. Comparisons were made between tears treated with sutures, tears treated with sutures soaked with adhesive and tears treated with only adhesive. The results showed best performance when the sutures were soaked with adhesive (~70 N), followed by sutures (~61 N) and adhesive (~60 N) only. This indicates the potential of this class of materials in meniscal repair.

Adhesives comprising isocyanate-terminated molecules have a similar bonding mechanism. They form a covalent bond with the tissue and cure to form polyurethane networks upon contact with the surrounding fluids [127], see Fig. 9b. Compared with other biomaterials, polyurethanes possess many advantages, such as high tenacity, toughness, water and chemical resistance and mechanical flexibility [128]. Biodegradable polyurethanes are designed to undergo (enzymatic) hydrolytic degradation to non-cytotoxic products [129]. Mostly aliphatic diisocyanates are used in the design of biomaterials intended for biomedical use, since it has been shown that they exhibit lower toxicity comparing to aromatic ones [130].

Hadba et al. described the development of two compositions of branched isocyanate-functional adhesives based on poly(ethylene glycol) and adipic acid functionalized with 2,4-toluene diisocyanate (TDI) or 4,40-methylene-bis(phenyl isocyanate) (MDI) [131]. The adhesive strength of the formulated materials to porcine stomach tissue depended on the specific formulation, the highest value determined was approximately 100 kPa (1093 gf/cm^2^).

Rohm et al. and Sternberg et al. [116, 132] reported the use of mixtures of 1,2-ethylene glycol bis-(dilactic acid) functionalized with hexamethylene diisocyanate (HDI) with different biodegradable polymers such as hyaluronic acid, gelatin, chitosan acetate and chitosan chloride. The obtained products had promising properties in terms of biodegradability, biocompatibility and adhesive strength (which was similar or better than fibrin glue). The solidification speed could be adjusted by using aqueous solution of chitosan chloride as a second component of the adhesive system.

Nayeb-Habib et al. developed a isocyanate pre-polymer and chitosan gel based tissue adhesive in order to combine good adhesion of isocyanates and biocompatibility and healing properties of chitosan [133]. Degradation products of this polymer did not induce any toxicity. Field et al. evaluated two isocyanate-based adhesives to repair full thickness meniscal wounds in an ovine model in vivo over a period of 1 month [134]. A 10 mm defect in the medial meniscus was repaired with these adhesives, and after 1 month signs of repair and tissue regeneration were observed and no adverse inflammatory reaction was present. Nevertheless, despite the progress in the development of less toxic materials with suitable mechanical properties and satisfactory curing times, until today there is only one isocyanate-based tissue adhesive commercially available: TissuGlu^®^ (Cohera Medicals Inc^®^). It is FDA-approved for use in abdominoplasty surgery.

#### Photo cross-linkable adhesives

Another interesting class of tissue adhesives are those that are photo cross-linkable, an example is commercially available FocalSeal. It is a copolymer composed of hydropihilic polyethylene glycol (PEG), and glycolide or lactide, trimethylene carbonate and acrylic acid moieties. These materials are crosslinked by light, which also initiates their adhesion to tissue [135]. The acrylate groups covalently bind to the amine groups of the tissue and form an interpenetrating network upon curing. The material is biodegradable; resorption takes approximately 3 months [97]. The application process, however, is complicated because delivery of a source of curing light to the application site can be technically difficult.

In another study Lang et al. developed acrylate-functionalized copolymers based on sebacic acid and glycerol that could be cross-linked with light [136]. The materials were used in vivo to attach a polymeric patch to the beating heart of rats and pigs. The patch remained in place even under higher than normal heart beat rates.

Ishihara et al. [137] worked on chitosan derivatives that could be crosslinked with UV light. The chitosan hydrogel obtained could stop bleeding from a cut mouse tail and keep two pieces of sliced mouse skin together. Moreover, application of the chitosan hydrogel induced significant wound contraction and accelerated wound closure and healing.

**Fig. 10:**
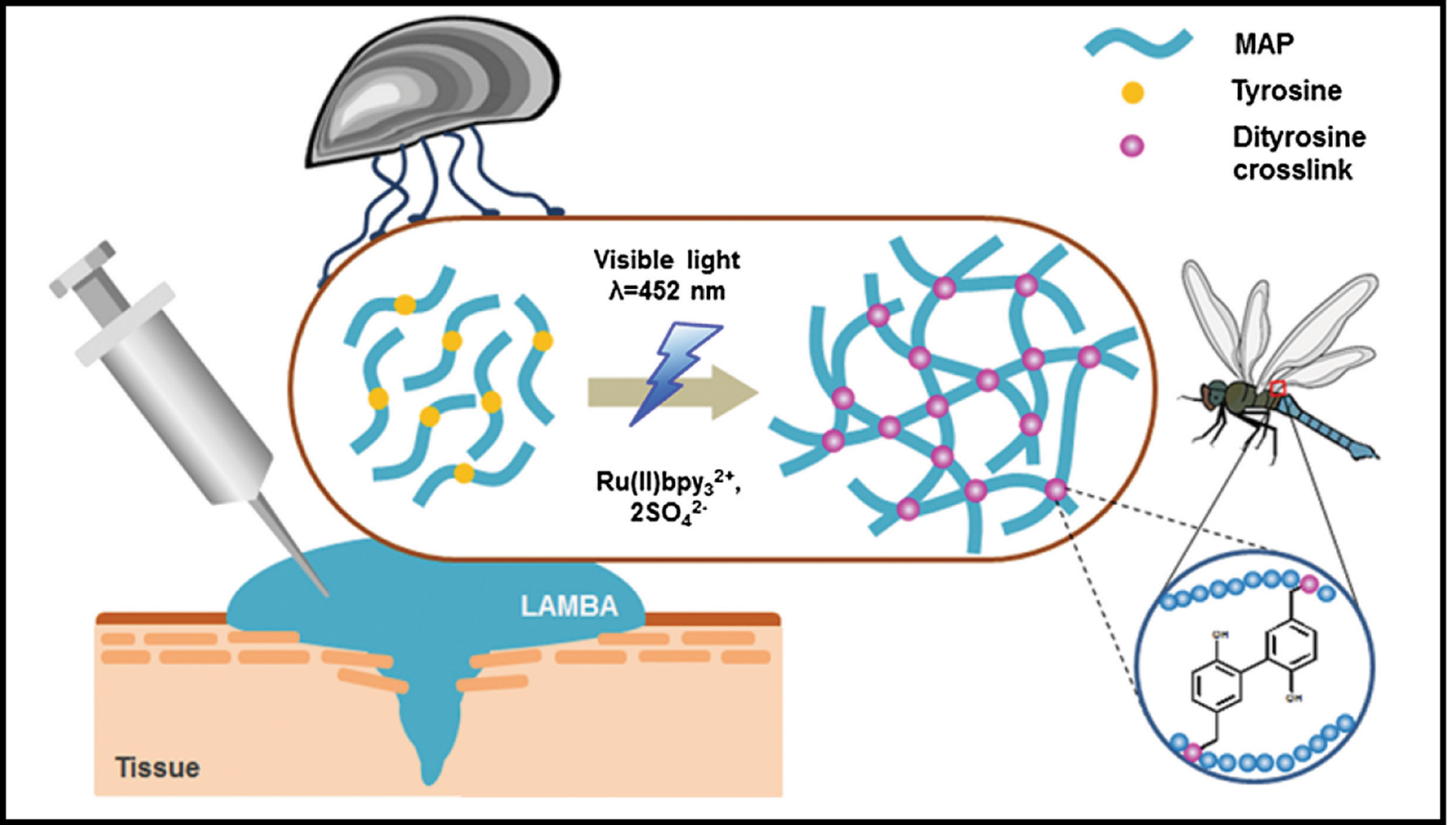
Network formation of a tissue adhesive with visible light using dityrosine groups (reprinted from [138])

In a recent study by Jeon et al. a light-activated adhesive based on mussel proteins and insect dityrosine crosslinking chemistry is reported [138]. The adhesive could be easily photo-cross-linked using visible light, the adhesion and cross-linking mechanism is depicted in Fig. 10. The adhesive strength to porcine skin was around 50 kPa, and no adverse tissue response after closing incisions on the back of rats was observed.

#### Thermo-responsive adhesives

A different group of materials are those, which can be cross-linked physically. Lando et al. and Cohn et al. developed an interesting approach, in which thermo-responsive biodegradable copolymers based on lactide-ε-caprolactone and three-armed trimethylolpropane-lactide-ε-caprolactone were used as tissue adhesives [139, 140]. These low molecular weight materials were in a flow state with low viscosity at the temperature of application and could attain high viscosity at the body temperature. The adhesive failure strength to polyamide (6,6-nylon) films, which was used as a model for a tissue, was measured to be up to 2 N/cm width of the film.

Although chemically modified tissue adhesives hold great potential for use in a variety of medical applications, further studies are required to develop materials for specific applications such as the treatment of meniscus tears.

### Formulation of tissue adhesives with biological factors

It has been recognized that incorporation of biological molecules, growth factors, platelet rich plasma (PRP), bone marrow (BM) and living cells to the adhesive can enhance healing of the tissue [86]. Ishimura et al. reported an experimental study in which they incorporated marrow cells into fibrin gel and compared the healing rate of the torn meniscus with fibrin gel that did not contain cells [85]. The results showed faster and more mature healing of defects in the group, which contained marrow cells. A similar approach was used by Scotti et al.: To improve the bonding between two swine meniscal slices, articular chondrocytes were added to a fibrin glue gel. The bonding capacity was evaluated in a nude mouse model after implantation for 4 weeks [141]. The results showed firm gross bonding in the experimental samples, while no bonding was observed in the control samples that did not contain cells. Although the success of this approach has only been described in a limited number of studies, the very promising results obtained justify the further investigation of cell-containing adhesives for meniscus repair.

## How do tissue adhesives compare with the requirements for repair of the damaged meniscus and the current gold standard treatment?

The most important prerequisites for a tissue adhesive to be suitable for meniscus tear repair include: adequate mechanical properties; suitable degradation profile, sufficient adhesive strength, short curing times and good biocompatibility (see Table 1). Comparing these requirements with the properties of the tissue adhesives described above, it can be concluded that up to now no tissue adhesive material has yet been developed that fully complies with all requirements. However, this not only due to sub-optimal properties of the adhesives, such as insufficient bonding strength in the case of enzymatically cross-linked adhesives or poor biocompatibility in the case of cyanoacrylates. It is also due to lack of the data that is required to be able to conclude if a certain material is suitable for the intended application. Simson et al., for example, described a promising bone marrow-chondroitin sulphate tissue adhesive for meniscus repair, however no information regarding its biodegradability was presented.

Moreover, although lap shear adhesion tests are one of the most often employed methods to assess the adhesive strength of newly developed glues, the conditions under which these tests are conducted are not standardized. There have been many substrates and tissue models used to evaluate the adhesion strength of tissue adhesives, e.g. polyamide films [139, 140], gelatin pieces [142–144], collagen membranes [96], and even pig skin and wood [73]. Additionally, very different environments (wet or dry), gluing temperature and the curing times have been used. These large variations make direct comparison of the outcomes of described experiments difficult.

Up to now, there have been only few clinical studies reporting the use of tissue adhesives in the repair of meniscal tears, all studies involved fibrin glue. As this particular adhesive has poor mechanical- and adhesive properties, it cannot be used as to replace the use of sutures as gold standard. There is much interest in the development of new adhesive materials. However, more systematic (clinical) research is needed before the suitability of a newly developed tissue adhesive for the intended application can be proven.

## Conclusions

Among the methods currently used to repair meniscus, the ideal solution has not yet been found. Tissue adhesives hold great potential to replace or support sutures and staples. Many new adhesive materials with a good prognosis for use in a variety of applications have been developed. However, most of them have not been sufficiently characterized to be able to qualify them as being suitable for meniscus repair. Nevertheless, chemically cross-linked adhesives seem the most versatile as these are based on existing natural or synthetic polymers and can be readily modified. Their properties can be adjusted by careful molecular design and chemical functionalization to make them suitable for the intended application.

Standardized relevant biomechanical and biological models need to be defined to compare different available and developed tissue adhesives, and to be able to address their suitability for the repair of meniscal tears. In clinical practice, the most successful treatment modality remains the use of sutures. Nevertheless, there is great need for a suitable meniscus tissue adhesive. Such adhesive should be easily applied in a meniscal tear, bind strongly to meniscal tissue, hold the torn region together, facilitate its healing, and then gradually degrade into non-toxic products.
